# Implementation of a non-communicable disease clinic in rural Sierra Leone: early experiences and lessons learned

**DOI:** 10.1057/s41271-021-00304-y

**Published:** 2021-09-08

**Authors:** Chiyembekezo Kachimanga, Yusupha Dibba, Marta Patiño, Joseph S. Gassimu, Daniel Lavallie, Santigie Sesay, Marta Lado, Alexandra V. Kulinkina

**Affiliations:** 1 Partners In Health, 25 Saquee Drive, Off Wilkinson Road, Freetown, Sierra Leone; 2 Harvard Medical School, 25 Shattuck Street, Boston, MA USA; 3 Ministry of Health and Sanitation, Wilkinson Road, Freetown, Sierra Leone; 4 Swiss Tropical and Public Health Institute, Socinstrasse 57, Basel, Switzerland; 5 University of Basel, Petersplatz 1, Basel, Switzerland

**Keywords:** Non-communicable disease, Hypertension, Diabetes, Rural health, Chronic disease care, Sierra Leone

## Abstract

This study is an evaluation of the first cohort of patients enrolled in an outpatient non-communicable disease clinic in Kono, Sierra Leone. In the first year, the clinic enrolled 916 patients. Eight months after the enrollment of the last patient, 53% were still active in care, 43% had been lost to follow-up (LTFU) and 4% had defaulted. Of the LTFU patients, 47% only came for the initial enrollment visit and never returned. Treatment outcomes of three patient groups [HTN only (*n* = 720), DM only (*n* = 51), and HTN/DM (*n* = 96)] were analyzed through a retrospective chart review. On average, all groups experienced reductions in blood pressure and/or blood glucose of approximately 10% and 20%, respectively. The proportions of patients with their condition controlled also increased. As NCDs remain underfunded and under-prioritized in low-income countries, the integrated program in Kono demonstrates the possibility of improving outpatient NCD care in Sierra Leone and similar settings.

## Key messages

- Health systems in the global south, primarily structured to address acute health problems, are underprepared to address the growing epidemic of non-communicable diseases.
- We demonstrate an example of an outpatient clinic and improvement in the outcomes of patients with hypertension and diabetes in the first year of its operation.
- We further recommend that NCDs are prioritized for funding and further integrated with existing longitudinal health services such as HIV.

## Introduction

Sierra Leone is a West African country with a population of 7.5 million [1]. It is among the poorest countries in the world, with over 60% of its residents living below the poverty line [2]. While its fragile health care system continues to recover from two major crises—an 11-year civil war and an unprecedented Ebola virus disease (EVD) outbreak—and reduce the burden of maternal mortality, malnutrition, malaria, pneumonia, and tuberculosis, non-communicable diseases (NCDs) are becoming another major epidemic [3].

According to recent estimates, NCDs account for approximately 33% of deaths in Sierra Leone, 14% attributed to cardiovascular diseases, 3% to cancers, 2% to chronic respiratory diseases, 2% to diabetes, and 12% to other conditions [4]. Furthermore, the Global Burden of Disease study identified NCD risk factors, such as poor diet, smoking, alcohol consumption, high blood pressure, body mass index (BMI), and blood glucose levels among the top ten risk factors for death and disability in the country [3]. Although NCD prevalence data is generally poor, it is estimated that 23% of Sierra Leoneans have raised blood pressure, 8% are obese, and 5% have raised blood glucose levels [4].

In recognition of the growing NCD epidemic, the Government of Sierra Leone through the Ministry of Health and Sanitation (MoHS) developed its first National NCD Policy and Strategic Plan in 2013 [5]. However, its implementation has been limited in the face of competing priorities, scarce resources, and lack of effective care delivery models [6]. A recent study reported that the majority of patients seek NCD services in the outpatient primary health setting [7]; yet in 2017 only 35% and 25% of community health centers offered cardiovascular disease and diabetes diagnosis and management services, respectively. Furthermore, most of these facilities were private and located in urban settings, with NCD service availability being much lower (7–15%) in public health facilities in rural areas [8].

As in many low- and middle-income settings, primary and secondary health care services in Sierra Leone are structured to support patients presenting primarily with acute conditions. The provision of high quality NCD services, on the other hand, requires a comprehensive and continuous care delivery model to allow for patient registration, assessment, treatment, counselling and longitudinal follow-up [9, 10]—a model similar to that of HIV. Due to similarities in management, integrating several chronic diseases, following the model of HIV, has proven efficient and effective in other resource-limited settings [11, 12].

Affordability presents another challenge for NCD care provision. In Sierra Leone, the MoHS waives user fees under the Free Healthcare Initiative for select populations, such as pregnant and lactating women, children under the age of 5 years, and other vulnerable groups including EVD survivors [13]. NCD patients are excluded from the free health care category, and are therefore expected to incur catastrophic out-of-pocket costs, as is the case in other low-income countries [14, 15]. For example, in rural Malawi, a chronic NCD condition accounts for 22% of monthly per capita expenditures [16]. In India, urban poor spend up to 34% of their income on diabetic care [17]. A high proportion of NCD patients in low-income countries, particularly women (38%), forego taking medications because of catastrophic expenditures pushing their households into poverty [14].

In light of the NCD epidemic in Sierra Leone, Partners In Health (PIH), an international non-governmental organization, in collaboration with the MoHS, established one of the first integrated NCD clinics at Koidu Government Hospital (KGH) in Kono district in 2018. Kono is located in the eastern part of the country, bordering Guinea, with a population of approximately 506,000, 75% of which live in rural areas [1]. The district is the former epicenter of the civil war and the heart of the ‘blood diamond’ industry, both of which have had significant negative economic, environmental, and health impacts. Mining continues to contribute to land erosion, water pollution, and destruction of agriculturally productive wetlands, deepening food insecurity [18]. The EVD outbreak also significantly affected the district, further undermining public trust in the already weak health system [19].

KGH is a public district hospital jointly operated by the MoHS and PIH. It is the only referral hospital in Kono, serving over 90 primary health care facilities distributed throughout the district. The ~ 200-bed facility provides inpatient services such as surgery, deliveries, and adult and pediatric admissions. Outpatient services include HIV and TB diagnosis and treatment, antenatal care, vaccinations, mental health clinic and a general outpatient department. In addition to the patient groups supported by the Free Healthcare Initiative, KGH provides free services to patients with physical disabilities, mental health disorders and other socially or economically disadvantaged persons through the partnership with PIH. Patients enrolled in the new outpatient integrated NCD clinic also received health services free of charge.

This study is an evaluation of the initial cohort of hypertensive and diabetic patients enrolled during the first year of the clinic’s operation, conducted through a retrospective chart review. The objectives of the study were to assess patient enrollment and outcomes after 8 to 20 months of follow-up and describe the challenges and lessons learned throughout the implementation process. Despite the increasing recognition of NCD mortality and morbidity in Sierra Leone, there is no published literature on the implementation, management and outcomes of integrated NCD programs. This study aims to inform future implementations of longitudinal NCD care in Sierra Leone and in similar resource-limited settings.

## Methods

### Clinic operation

**Fig. 1:**
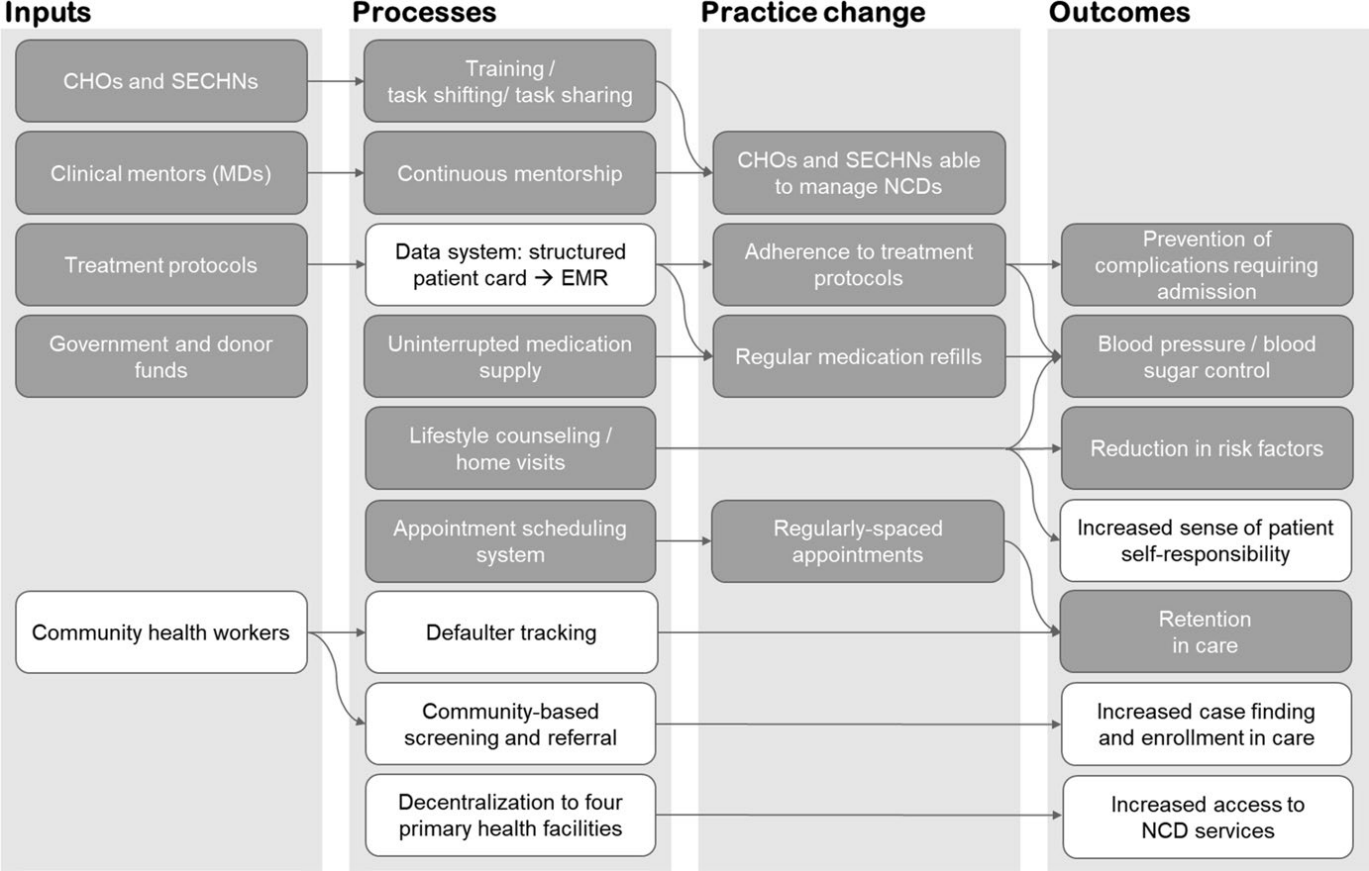
Logic model of the non-communicable disease (NCD) program. Dark gray boxes represent elements that were present in the first year of the clinic’s operation; white boxes were only partially present or aspirational for the subsequent years of the program. *CHO* Community health officer, *SECHN* state enrolled community health nurse, *MD* medical doctor, *EMR* electronic medical record

The KGH NCD clinic was established in February of 2018 and initially focused on patients with hypertension (HTN) and diabetes mellitus (DM). The clinic was operated primarily by community health officers (CHO) and state enrolled community health nurses (SECHN) under the mentorship of medical doctors (MD) and organized under the task-shifting model. SECHNs registered patients, took and recorded demographic information and basic clinical measurements in patient charts, and conducted lifestyle counseling. Initially, MDs conducted clinical consultations, which included medical history, physical examination, ordering laboratory tests (if needed), and prescribing medications. Overtime, stable patients were handed over to the CHOs, while MDs focused on more clinically complex cases. The clinic was staffed by a combination of MoHS and PIH health workers, but overseen and continuously mentored by PIH doctors. Laboratory tests and dispensing of medications were integrated into the normal lab and pharmacy systems, but financed by PIH. The PIH monitoring and evaluation team provided clerical and data systems support. A limited logic model for the integrated outpatient NCD program is shown in Fig. 1.

### Patient enrollment

Patients were enrolled in care if they had been previously diagnosed (in another location) and already on treatment, previously admitted to KGH due to complications from one of these conditions, or after initial screening conducted on specific clinic days. New HTN patients based on the initial screening were enrolled in care if they had two blood pressure measurements ≥ 140/90 mmHg taken at least two minutes apart. New DM patients were enrolled if they had a random blood glucose level ≥ 11.1 mmol/L and diabetes symptoms (e.g. increased thirst, urination, fatigue, blurred vision) or a fasting blood glucose level ≥ 7 mmol/L.

### Patient management

During registration, patients received unique identifiers linked with clinical charts, which were maintained and updated throughout their care. During the first visit, CHOs and SECHNs took and recorded demographics and basic clinical measurements, including age, sex, height, weight, blood pressure, and blood glucose level. A clinical consultation followed. Follow-up appointments were scheduled based on the individual patients’ condition. During follow-up appointments, patients’ clinical charts were updated with new basic measurements, clinical notes and laboratory test results, and their prescriptions were refilled free of charge. CHOs and SECHNs also conducted lifestyle modifications counselling via individual and group health talks, focused on dietary advice (including reduction of salt and alcohol intake) and regular exercise.

In 2019, the MoHS was in the process of developing NCD treatment guidelines and job aids for use in the government health facilities. We adapted the treatment guidelines from the draft MoHS documents, supplemented by the HEARTS technical package [20]. For HTN treatment, a stepwise approach was used with thiazide (mainly hydrochlorothiazide), calcium channel blockers (mainly Amlodipine), angiotensin-converting enzyme (ACE) inhibitors (mainly Enalapril) and beta-blockers (mainly Atenolol) as steps 1, 2, 3 and 4, respectively. For type 2 DM, the first line treatment was Metformin, with Glibenclamide added as step 2. In cases where patients failed to respond to both medications at maximum doses, they were started on insulin. For type 1 DM, CHOs and SECHNs were trained on insulin management. Initially (in the first 2–3 weeks), these patients were seen twice daily (mostly in the clinic, but in some cases at home) to ensure they know how to self-administer insulin correctly and gauge the best dose to control their blood glucose. Subsequently, they were seen monthly. Initially, insulin vials (short- and intermediate-acting) were used, but due to challenges with storage in patients’ homes (i.e. lack of a refrigerator and stable power supply), we later switched to insulin pre-filled pens (Apidra® and Lantus®).

### Inclusion criteria and variable definitions

We initially included all patients (*N* = 982) who enrolled in care within the first year of the clinic’s operation (between February 1, 2018 and January 31, 2019). However, 66 patients were considered as ‘discharged’ and excluded in the analysis stage because they had only come to the clinic once (self-referred), had normal blood pressure and blood glucose levels, never received a diagnosis or medications and did not return. The total resulting cohort of 916 patients was analyzed.

To obtain the analysis variables, we conducted a retrospective chart review in October 2019, meaning that each included patient was under observation for a minimum of eight months (February 1 to September 30, 2019) and a maximum of 20 months (February 1, 2018 to September 30, 2019). Baseline and follow-up data were abstracted from the clinical charts to describe enrollment characteristics and evaluate retention in care and blood pressure and blood glucose control of HTN and DM patients, respectively. Baseline and follow-up measurements refer to measurements recorded at the first and last visit within the study period, respectively. The primary outcomes in the analysis were retention in care and good control of the condition. Other analysis variables included routine metrics recorded in the clinical charts (Table 1).

**Table 1:** Summary of analysis variables

Outcome 1: Condition control
● For patients with hypertension (HTN): blood pressure < 140/90 mmHg ● For patients with diabetes mellitus (DM): random blood glucose level < 7 mmol/L
Outcome 2: Retention in care
● Active: attended last scheduled appointment or less than 30 days past last scheduled appointment ● Defaulted: 30–90 days elapsed since a missed appointment ● Lost to follow-up (LTFU): 90 or more days elapsed since missed appointment
Demographic and clinical variables
● Age (years): recorded at baseline only and categorized as follows: < 25 years; 25–44 years; 45–64 years; 65 + years ● Sex (male or female): recorded at baseline only ● Height (m): recorded at baseline only ● Weight (kg): recorded at baseline and follow-up ● Body mass index (BMI) (kg/m^2^): calculated at baseline and follow-up and categorized as follows: < 18.5 (underweight); 18.5–24.9 (normal); 25–29.9 (overweight); 30 + (obese) ● Blood pressure (mmHg): recorded at baseline and follow-up ● Fasting or random blood glucose (mmol/L): recorded at baseline for all patients and at follow-up for DM patients only ● Treatment duration (weeks): calculated as the difference between last visit date and enrollment date

### Data analysis

Data analysis included descriptive statistics of the patient population at baseline and follow-up. We evaluated change in continuous measurements (e.g. systolic or diastolic blood pressure) between baseline and follow-up using a paired sample t-test, with statistical significance determined by a p-value < 0.05. The effects of demographic and clinical measurements on the binary primary outcomes (LTFU and blood pressure or blood glucose control status) were assessed using logistic regression. Data analysis was conducted using R software (version 3.6.1).

We tested five available variables as predictors of blood pressure and blood glucose control: sex, age category, BMI category at baseline, controlled blood pressure or blood glucose at baseline, and treatment duration. The same variables, plus controlled blood pressure or blood glucose at follow-up, minus treatment duration were tested as predictors of LTFU in the full cohort (Table 1).

### Ethical approval

The Sierra Leone’s Office of Ethics and Scientific Review Committee approved this study. Informed consent was waived because all data were collected through routine clinical care and de-identified before being retrospectively analyzed.

## Results

### Enrollment, demographics and outcomes

#### Enrollment and distribution of diagnoses

**Fig. 2:**
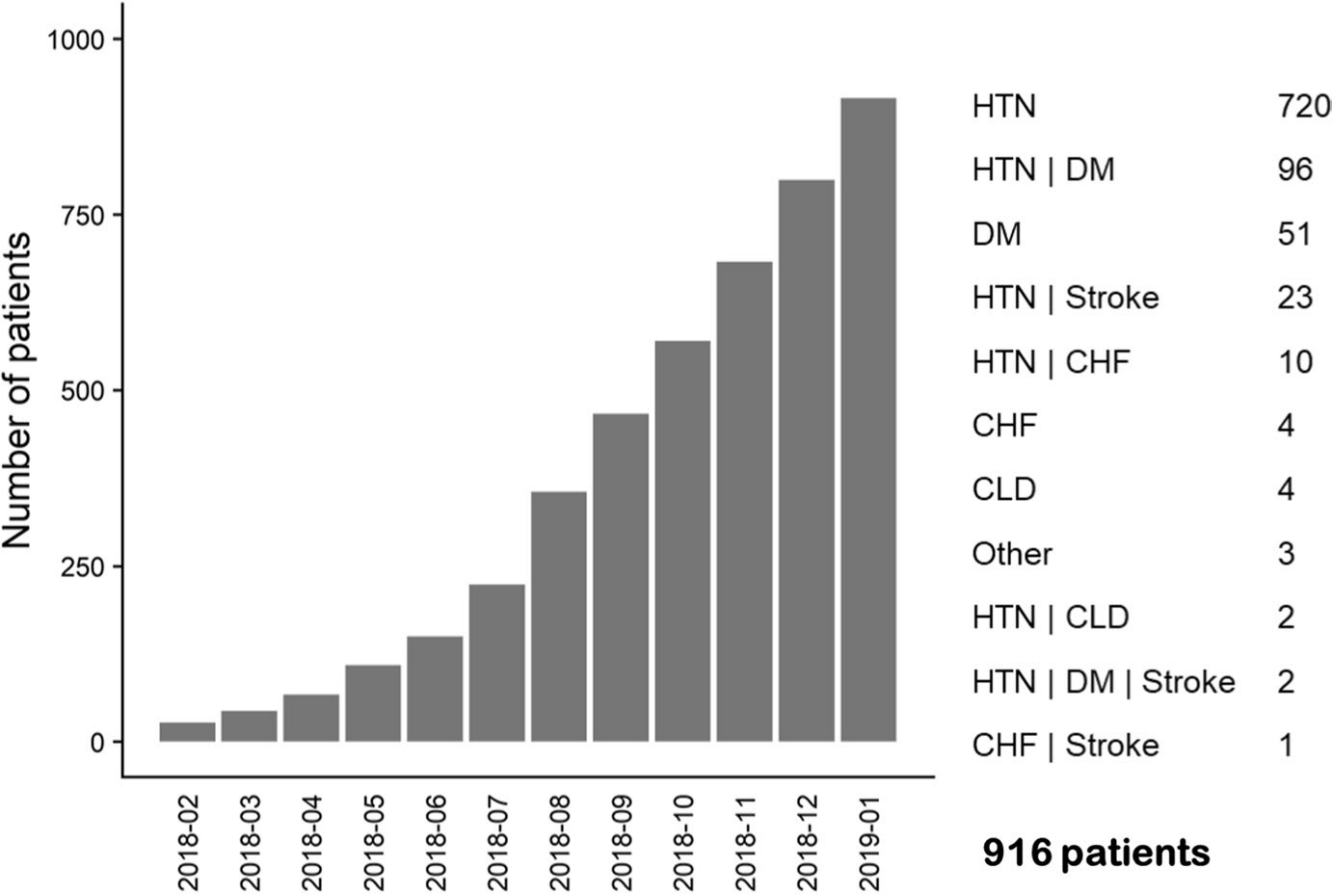
Overall cohort enrollment and distribution of diagnoses: hypertension (HTN); diabetes mellitus (DM), stroke, congestive heart failure (CHF); chronic liver disease (CLD), and others

In the first year of operation, the clinic enrolled a total of 916 clients (Fig. 2). Initially only HTN and/or DM patients were enrolled, with other chronic conditions such as congestive heart failure (CHF), stroke, and chronic liver disease (CLD) diagnosed and added over time. As such, at the end of the first year, the majority of the cohort (93%) had HTN diagnosis, either alone (720) or in combination with other comorbidities (133). A total of 149 patients had DM, either alone (51) or in combination with HTN (96) or HTN and stroke (2). Of the DM patients, the majority (90%) had type 2 DM. At the end of the first year, only 13 patients had other chronic diseases without HTN or DM as comorbidities (Fig. 2), including three patients with nephrotic syndrome and gestational HTN that were classified as “other”.

#### Characteristics at baseline

**Fig. 3:**
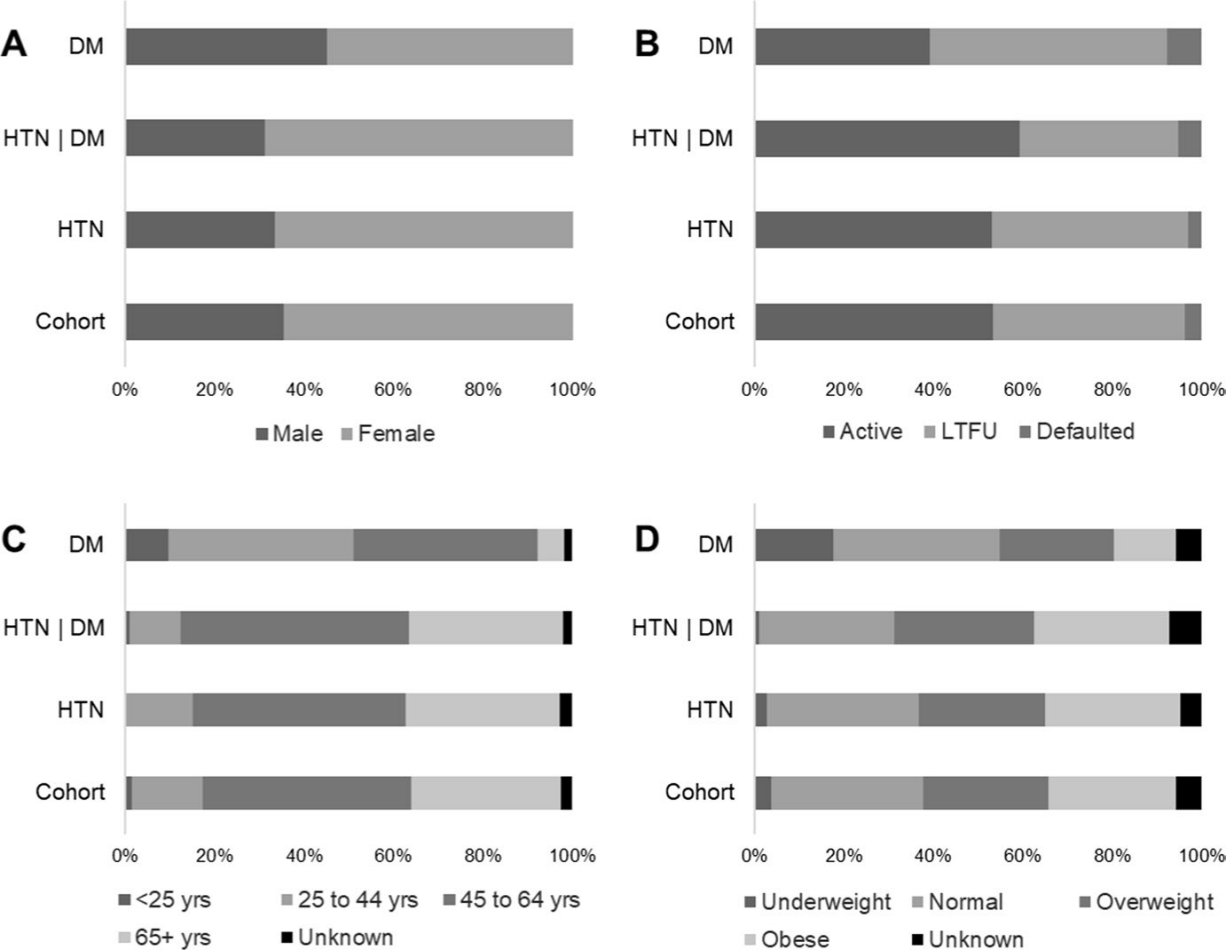
Distribution of sex (**a**), outcome (**b**), age (**c**) and body mass index (**d**) among the entire cohort, patients with hypertension (HTN) or diabetes mellitus (DM) only, and patients with HTN/DM comorbidities. Lost to follow-up (LTFU)

The majority of the overall cohort were female (65%), older than 45 years of age (80%), and overweight or obese (56%) at enrollment. Because the majority of the cohort was composed of HTN patients, the characteristics of HTN and HTN/DM patient subgroups were similar to the overall cohort (Fig. 3, Table 2). DM patients, however, were more balanced in terms of sex distribution, on average 13 years younger (95% CI: 8.7, 17.0) and had 3.5 units lower BMI (95% CI: 1.6, 5.1) at baseline as compared to the overall cohort.

**Table 2:** Summary of analysis variables (difference in the total cohort size and the sum of HTN, DM, and HTN/DM patients is accounted for by 49 patients with other conditions)

	Cohort	HTN	HTN | DM	DM
	*N* = 916	*N* = 720	*N* = 96	*N* = 51
Values at enrollment				
Sex				
Sex recorded	916	720	96	51
Males	325	241	30	23
Females	591	479	66	28
Age				
Age recorded	892	700	94	50
> 25 years	14	3	1	5
25–44 years	145	106	11	21
45–64 years	427	342	49	21
65 + years	306	249	33	3
Body mass index				
Height and weight recorded	863	686	89	48
Underweight	35	21	1	9
Normal	312	244	29	19
Overweight	255	203	30	13
Obese	261	218	29	7
Blood pressure				
Blood pressure recorded	888	702	92	49
Average systolic blood pressure	166 (± 31)	171 (± 29)	163 (± 28)	118 (± 18)
Average diastolic blood pressure	97 (± 18)	99 (± 17)	96 (± 15)	76 (± 10)
Blood pressure control	–	44	9	–
Blood sugar				
Blood sugar recorded	866	680	92	50
Average blood sugar	6.9 (± 4.5)	5.3 (± 1.5)	13.8 (± 5.9)	16.5 (± 6.6)
Blood sugar control	–	–	9	3
Values at follow-up				
Body mass index				
Weight recorded	566	449	64	22
Underweight	29	24	0	3
Normal	224	174	22	9
Overweight	156	122	23	8
Obese	157	129	19	2
Blood pressure				
Blood pressure recorded	722	558	83	38
Average systolic blood pressure	147 (± 27)	149 (± 27)	146 (± 25)	130 (± 26)
Average diastolic blood pressure	90 (± 15)	90 (± 15)	91 (± 14)	84 (± 12)
Blood pressure control	–	174	23	–
Blood sugar				
Blood sugar recorded	143	30	76	33
Average blood sugar	10.1 (± 5.4)	6.3 (± 2.2)	10.8 (± 5.5)	12.4 (± 5.5)
Blood sugar control	–	–	19	6
Outcome				
Duration on treatment (weeks)	34.9 (± 18.5)	34.6 (± 18.1)	36.7 (± 18.6)	34.2 (± 22.1)
Active	489	382	57	20
Lost to follow-up	393	316	34	27
Defaulted	34	22	5	4

#### Outcomes

At the end of the observation period, approximately half of the first year cohort (53%) were still active in care, 43% had been LTFU, and 4% had defaulted (Fig. 3). Average treatment duration for the overall cohort was approximately 35 (± 18.5) weeks, with no difference among the patient subgroups (Table 2). After excluding LTFU and defaulted patients, treatment duration increased to 43.8 (± 13.1) weeks. Of the 393 LTFU patients, 185 (47%) only came for the initial enrollment visit and never returned. Among the remaining LTFU patients, the average treatment duration was 14.2 (± 12.3) weeks. Patients who defaulted had an average treatment duration of 32.5 (± 15.3) weeks. There were no significant differences in LTFU rates among the patient subgroups.

### Changes in patient condition between baseline and follow-up

#### Hypertensive patients

**Fig. 4:**
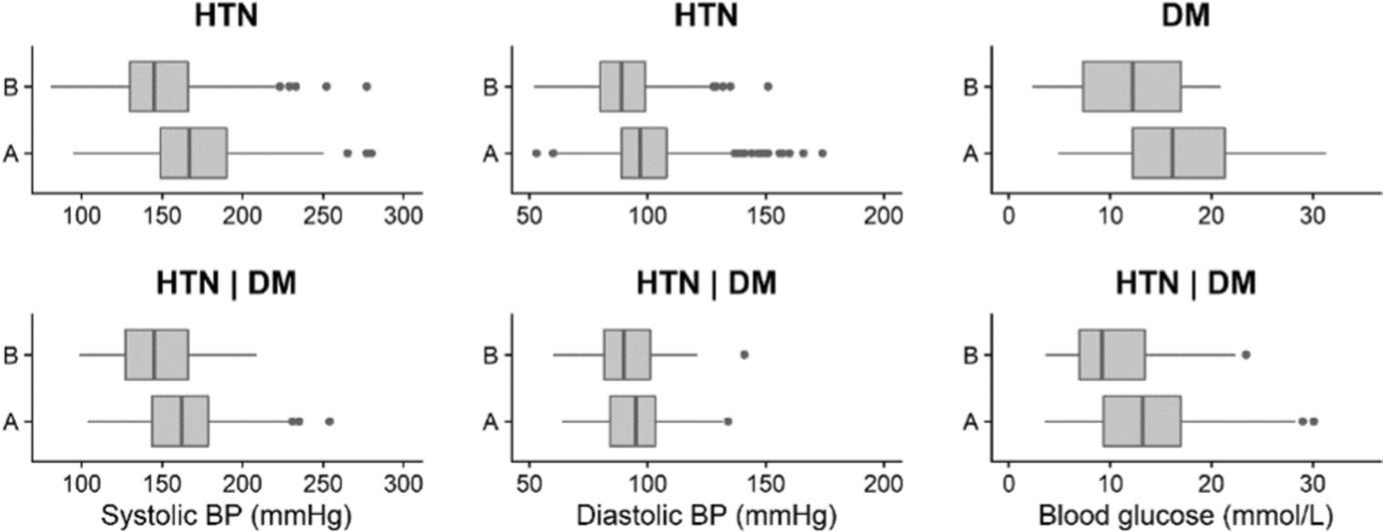
Boxplots of systolic and diastolic blood pressure (BP) and random blood glucose measurements at baseline (**a**) and follow-up (**b**) for the hypertension (HTN), diabetes mellitus (DM) and HTN/DM patient subgroups

A total of 720 patients in the cohort had HTN diagnosis only. The average systolic blood pressure (SBP) and diastolic blood pressure (DBP) at baseline for these patients were approximately 171 (± 29) and 99 (± 17) mmHg, respectively. At follow-up, both values decreased to 149 (± 27) and 90 (± 15) mmHg, respectively (Fig. 4, Table 2). The percentage of patients with controlled blood pressure increased from approximately 6% at baseline to 31% at follow-up (*p* < 0.001). According to a paired sample t-test conducted on the smaller subgroup of patients who had blood pressure measurements at baseline and follow-up (*n* = 541), on average SBP and DBP decreased by 23.4 mmHg (*p* < 0.001) and 9.5 mmHg (*p* < 0.001), respectively. Additionally, the BMI of the HTN patients decreased slightly between baseline and follow-up visits by 1.1 units (*p* < 0.001).

#### Diabetic patients

A total of 51 patients in the cohort had DM diagnosis only. The average random blood glucose level at baseline for these patients was approximately 16.5 (± 6.6) mmol/L and decreased to 12.4 (± 5.5) mmol/L at follow-up (Fig. 4, Table 2). The percentage of patients with controlled blood glucose increased from approximately 6% at baseline to 18% at follow-up but was not statistically significant due to a lower number of patients who had a follow-up measurement recorded as compared to baseline. According to a paired sample t-test conducted on a smaller subgroup of patients who had blood glucose measurements at baseline and follow-up (*n* = 33), on average, blood glucose level decreased by approximately 3 mmol/L (*p* < 0.05). No change in the BMI of the DM patients was observed between baseline and follow-up.

#### Patients with comorbidities

A total of 96 patients in the cohort had HTN and DM comorbidities. The average SBP and DBP at baseline for these patients were lower than for HTN patients only at approximately 163 (± 28) and 96 (± 15) mmHg, respectively. At follow-up, both values decreased to 146 (± 25) and 91 (± 14) mmHg, respectively (Fig. 4, Table 2). The percentage of patients with controlled blood pressure increased from approximately 10% at baseline to 28% at follow-up (*p* < 0.01). According to a paired sample t-test on patients who had blood pressure measurements at baseline and follow-up (*n* = 78), on average SBP and DBP decreased by 19.6 mmHg (*p* < 0.001) and 5.0 mmHg (*p* < 0.05), respectively.

The average random blood glucose level at baseline for these patients was lower than of patients who had DM diagnosis only, at approximately 13.8 (± 5.9) mmol/L, which decreased to 10.8 (± 5.5) mmol/L at follow-up (Fig. 4, Table 2). The percentage of patients with controlled blood glucose increased significantly from approximately 10% at baseline to 25% at follow-up (*p* < 0.05). According to a paired sample t-test conducted on a smaller subgroup of patients who had blood glucose measurements at baseline and follow-up (*n* = 71), on average, blood glucose level decreased by approximately 3 mmol/L (*p* < 0.01). No change in the BMI of HTN/DM patients in this sub-group was observed between baseline and follow-up.

### Predictors of controlled condition and outcome

Only controlled blood pressure at baseline was a significant predictor of controlled blood pressure at follow-up in a univariable model (OR 2.36; 95% CI: 1.26, 4.41), and only for the HTN but not for the HTN/DM patients. None of the tested variables were significantly associated with blood glucose control in any of the patient subgroups. HTN patients who had controlled blood pressure at baseline were also less likely to be LTFU at the end of the observation period as compared to those who enrolled in care with their blood pressure uncontrolled (OR 0.40; 95% CI: 0.19, 0.78). This association was only observed in the HTN patient population but not in the other subgroups.

## Discussion

In the first year of its operation, the NCD clinic grew very rapidly, enrolling over 900 patients. The cohort was comprised of predominantly patients with HTN, women, individuals over 45 years of age, and those classified as overweight or obese according to their BMI. Furthermore, most patients (> 90%) were enrolled in care with their condition uncontrolled. These observations support the underlying assumption that in the early stages of service provision, the target population is that with severe symptoms, prominent risk factors, and better health seeking behaviors. From the limited location data recorded in the patient files, it is also true that in the first year, the clinic was reaching largely the urban and peri-urban population of Koidu town, with fewer patients coming from the district’s interior.

According to the most recent census, 506,000 people reside in Kono district, 230,000 adults aged 18 + [1]. At the current WHO estimates of 23% HTN and 5% DM prevalence rates among adults [4], there are approximately 53,000 hypertensive and 11,500 diabetic individuals living in Kono. The respective numbers (for HTN and DM) for Koidu town are 14,000 and 3000, meaning that the clinic is currently serving only 5–7% of the potential target population of HTN and/or DM patients in Koidu town, and a much smaller proportion of Kono district overall.

A recent nationally representative population-based study in Kenya showed that only 15% of individuals with HTN were aware of their elevated blood pressure and only 27% of those who were aware were on treatment [21]. At the current enrollment numbers, the clinic has already reached those “low hanging fruit” patients with severe illness and/or good health seeking behaviors. For minor symptoms, people tend to avoid the health care system and self-refer to herbalists and traditional medicine vendors [6]. It is important, however, to reach these patients early, before complications arise, through improving knowledge about NCD services in the district, decentralizing service provision to the primary health care level, and conducting blood pressure and blood glucose screening in communities.

In terms of treatment effects, the program demonstrated success among all patient subgroups. Percentage of patients with controlled blood glucose increased from approximately 8% to 22% over an average treatment duration of 36 weeks, considering DM and HTN/DM patients. Other studies conducted in specialized diabetic clinics showed higher rates of glycemic control of approximately 40%; however, these were often patients who had been on treatment for five years or more [22] or had used a home glucometer [23]. The 22–25% reduction in average random blood glucose measurements was two times higher than in another study over a similar observation period; although the baseline average blood glucose in our study was also higher (13–16 mmol/L vs. 10 mmol/L) [24]. Percentage of patients with controlled blood pressure also increased from approximately 7% to 31% over an average treatment duration of 35 weeks, considering patients with HTN only and those with comorbidities. Rates of blood pressure control were similar or lower in other studies, typically in the rage of 23–27% [25–27].

The relatively high LTFU rate (around 40%) in our study was similar to those reported in other early NCD program implementations [12, 28]. Importantly, nearly half of all LTFU patients had only come for the initial enrollment visit and never returned. Due to a lack of home-based follow-up, it was impossible to know if the LTFU patients had died, moved out of the district, or stopped coming to the clinic for another reason. For example, many of the HTN patients (57 of 158) who came only for the enrollment visit had elevated but relatively low blood pressure (SBP < 160 and DBP < 100). Although the clinic is free, these patients may not have felt ‘sick enough’ to necessitate the payment of the transportation costs and/or lose a full day of wages to come for their clinic appointments. Conversely, a smaller number of these patients (12 of 158) had very high blood pressure (SBP > 200 and DBP > 100) and may have died. This finding presents a strong argument for prioritizing patients with uncontrolled condition for intensive adherence counseling and a home visit or at minimum a phone call if they miss their early follow-up appointment.

Over the first year of the clinic’s operation, many challenges were identified. For example, the rapid increase in patient numbers meant shortages of space, staff, and medications. The small consultation room and few clinicians allotted to the clinic for one day per week meant that patients waited in long queues, were seen in crowded spaces with limited privacy, and had shorter and less frequent appointments than desired. Over time, the clinic was moved to a larger space, increased its staffing, and began operating with increased frequency (two days per week) in order to meet the demand.

Another significant challenge is the lack of home-based follow-up to address high LTFU rates. This can be addressed by integrating with the existing community health worker (CHW) program that currently assists HIV and TB patients with medication adherence and accompaniment to the health facility for medication refills, laboratory tests, and clinical assessments. More generally, NCDs could be similarly embedded into the HIV care provision platform as part of an integrated chronic care model. This model has been successfully implemented in several instances, including by PIH in Neno district in Malawi [12] and as part of the SEARCH study in rural Uganda [29].

An additional area of improvement and integration is the patient record. The existing unstructured paper-based data system consisting primarily of unstructured clinical notes resulted in limited data that could be manually abstracted and analyzed in this study. The use of a single follow-up measurement, resulting in different treatment durations for each patient in the study was a limitation in the analysis. Although we confirmed that treatment duration did not significantly affect any of the variables we analyzed, at minimum, a structured patient card that systematizes the data collected at each visit for a specific condition would allow for more interesting and robust analyses, including multiple observations per patient. In fact, shortly after this analysis, a combined HTN/DM patient card modeled from those used by PIH in Malawi and Liberia, was introduced.

Beyond the patient card, an electronic medical record (EMR) is the ultimate goal for any such longitudinal care program. In many countries like Sierra Leone, in the context of vertically-funded programs, patients can be seen in different physical spaces, by different health care providers, and on different clinic days for multiple conditions. An EMR can link the records together and enable holistic treatment of the patient with his/her health information available to all health care providers. Data systems, although rarely seen as such, can underpin the integration of multiple health services for a patient-centered approach.

## Conclusion

Despite some progress in recent years, NCDs remain underfunded and under-prioritized by health systems of low-income countries that remain focused on episodic care and relief of acute symptoms with weak links up the referral pathway to specialized care and down to communities [9]. Chronic disease management requires a comprehensive approach that spans a range of interventions, such as primary prevention, case finding, community education, and treatment models that enable continuity of care [30]. Health delivery systems of low-income countries lack most of these components outside of HIV programs. Rather than re-inventing the wheel, we must capitalize on the gains achieved in HIV to establish continuity of care, such as appointment systems, medical records, counseling and adherence support, longitudinal monitoring and evaluation systems, and community outreach [30].

We showed in this manuscript that an integrated NCD model can work in rural Sierra Leone as part of an NGO-supported program. The PIH model, built on the premise of high-quality free health care, overcomes several barriers, namely lack of financial means to seek care, non-functioning equipment, and an unstable supply of quality drugs [28]. The next steps are to decentralize the program to primary health care facilities and to integrate community health workers into the NCD care model (Fig. 1). At present, the model requires substantial and consistent financing. In the short term, evidence from a successful donor-supported program can facilitate funding acquisition to scale the program. In the longer term, increasing commitment to addressing the NCD burden from the government will be needed to facilitate integration of chronic care into the primary health care model and to provide patients with affordable and continuous NCD care for life.

## References

[1] Population and Housing Census 2015. Statistics Sierra Leone. 2016. https://www.statistics.sl/index.php/census/census-2015.html. Accessed 12 Oct 2020.

[2] About Sierra Leone. United Nations Development Programme. 2020. https://www.sl.undp.org/content/sierraleone/en/home/countryinfo.html. Accessed 20 Dec 2020.

[3] Sierra Leone, Institute for Health Metrics and Evaluation. 2019. http://www.healthdata.org/sierra-leone. Accessed 5 Nov 2020.

[4] Noncommunicable Diseases Country Profiles. World Health Organization. 2018. https://www.who.int/nmh/publications/ncd-profiles-2018/en/. Accessed 5 Nov 2020.

[5] National Non-Communicable Diseases Strategic Plan 2013–2017. Ministry of Health and Sanitation. 2013. http://www.iccp-portal.org/system/files/plans/Sierra%20Leone%20-%20NCD%20strategic%20plan%20doc%202013-2017.pdf. Accessed 1 Dec 2020.

[6] Witter S, Zou G, Diaconu K, Senesi RGB (2020). Opportunities and challenges for delivering non-communicable disease management and services in fragile and post-conflict settings: perceptions of policy-makers and health providers in Sierra Leone. Confl Health.

[7] Koroma IB, Javadi D, Hann K, Harries AD (2019). Non-communicable diseases in the Western Area District, Sierra Leone, following the Ebola outbreak. F1000research.

[8] Sierra Leone SARA Plus Report 2017. Ministry of Health and Sanitation. 2019. https://www.opendatasl.gov.sl/dataset/sierra-leone-sara-plus-report-2017. Accessed 1 Dec 2020.

[9] Kruk ME, Nigenda G, Knaul FM (2015). Redesigning primary care to tackle the global epidemic of noncommunicable disease. Am J Public Health.

[10] Kumar A, Schwarz D, Acharya B, Agrawal P (2019). Designing and implementing an integrated non-communicable disease primary care intervention in rural Nepal. BMJ Glob Health.

[11] Njuguna B, Vorkoper S, Patel P, Reid MJA (2018). Models of integration of HIV and noncommunicable disease care in sub-Saharan Africa: lessons learned and evidence gaps. AIDS.

[12] Wroe EB, Kalanga N, Mailosi B, Mwalwanda S (2015). Leveraging HIV platforms to work toward comprehensive primary care in rural Malawi: the Integrated Chronic Care Clinic. Healthcare.

[13] National Health Sector Strategic Plan 2017–2021. Ministry of Health and Sanitation. 2017. https://extranet.who.int/countryplanningcycles/sites/default/files/planning_cycle_repository/sierra_leone/sierra_leone_nhssp_2017-21_final_sept2017.pdf. Accessed 1 Nov 2020.

[14] Murphy A, Palafox B, Walli-Attaei M, Powell-Jackson T (2020). The household economic burden of non-communicable diseases in 18 countries. BMJ Glob Health.

[15] Impact of out-of-pocket payments for treatment of non-communicable diseases in developing countries: A review of literature. World Health Organization. 2011. https://www.who.int/health_financing/documents/cov-dp_e_11_02-ncd_finburden/en/. Accessed 24 Nov 2020.

[16] Wang Q, Fu AZ, Brenner S, Kalmus O (2015). Out-of-pocket expenditure on chronic non-communicable diseases in Sub-Saharan Africa: The case of rural Malawi. PLoS ONE.

[17] Ramachandran A, Ramachandran S, Snehalatha C, Augustine C (2007). Increasing expenditure on health care incurred by diabetic subjects in a developing country: a study from India. Diabetes Care.

[18] Wilson SA. Diamonds, a resource curse? The case of Kono District in Sierra Leone. PhD Dissertation. Michigan State University. 2010.

[19] Elston JWT, Moosa AJ, Moses F, Walker G (2016). Impact of the Ebola outbreak on health systems and population health in Sierra Leone. J Public Health.

[20] HEARTS: Technical package for cardiovascular disease management in primary health care. World Health Organization. 2018. https://www.who.int/cardiovascular_diseases/hearts/en/. Accessed 3 Mar 2021.

[21] Mohamed SF, Mutua MK, Wamai R, Wekesah F (2018). Prevalence, awareness, treatment and control of hypertension and their determinants: results from a national survey in Kenya. BMC Public Health.

[22] Yigazu DM, Desse TA (2017). Glycemic control and associated factors among type 2 diabetic patients at Shanan Gibe Hospital. Southwest Ethiopia BMC Res Notes.

[23] Mideksa S, Ambachew S, Biadgo B (2018). Glycemic control and its associated factors among diabetes mellitus patients at Ayder comprehensive specialized hospital. Mekelle-Ethiopia Adipocyte.

[24] Pastakia SD, Nuche-Berenguer B, Pekny CR, Njuguna B (2018). Retrospective assessment of the quality of diabetes care in a rural diabetes clinic in Western Kenya. BMC Endocr Disord.

[25] Horsa BA, Tadesse Y, Engidawork E (2019). Assessment of hypertension control and factors associated with the control among hypertensive patients attending at Zewditu Memorial Hospital: a cross sectional study. BMC Res Notes.

[26] Antignac M, Diop IB, de Terline DM, Kramoh KE (2018). Socioeconomic status and hypertension control in sub-Saharan Africa: The multination EIGHT study (evaluation of hypertensio in sub-Saharan Africa). Hypertension.

[27] Ssinabulya I, Nabunnya Y, Kiggundu B, Musoke C (2016). Hypertension control and care at Mulago Hospital ambulatory clinic. Kampala-Uganda BMC Res Notes.

[28] Zou G, Witter S, Caperon L, Walley J (2020). Adapting and implementing training, guidelines and treatment cards to improve primary care-based hypertension and diabetes management in a fragile context: results of a feasibility study in Sierra Leone. BMC Public Health.

[29] Kwarisiima D, Atukunda M, Owaraganize A, Chamie G (2019). Hypertension control in integrated HIV and chronic disease clinics in Uganda in the SEARCH study. BMC Public Health.

[30] Rabkin M, El-Sadr WM (2011). Why reinvent the wheel? Leveraging the lessons of HIV scale-up to confront non-communicable diseases. Glob Public Health.

